# Supplementary material for: Behavioral Voluntary and Social Bioassays Enabling Identification of Complex and Sex-Dependent Pain-(-Related) Phenotypes in Rats with Bone Cancer
Source: Cancers (Basel). 2023 Mar 2;15(5):1565. doi: 10.3390/cancers15051565 (PMC10000428; doi:10.3390/cancers15051565)
Supplement: Supplementary file 1 [file cancers-15-01565-s001.zip › cancers-2147643-supplementary.pdf]

Table S1. Cluster analysis of PCA results (Figure 2) by multivariate analysis of variance (MANOVA).

Time	Model	♀/♂	Sig	Wilks-Lambda
Pre	Naive/Sham	♀	0.867	0.969
Pre	Naive/CIBP	♀	0.567	0.902
Pre	Sham/CIBP	♀	0.356	0.853
3d	Naive/Sham	♀	0.340	0.787
3d	Naive/CIBP	♀	0.776	0.955
3d	Sham/CIBP	♀	0.41	0.872
6d	Naive/Sham	♀	0.479	0.849
6d	Naive/CIBP	♀	0.142	0.457
6d	Sham/CIBP	♀	0.211	0.553
8d	Naive/Sham	♀	0.616	0.898
8d	Naive/CIBP	♀	<.001***	0.045
8d	Sham/CIBP	♀	<.001***	0.064
14d	Naive/Sham	♀	0.777	0.946
14d	Naive/CIBP	♀	<.001***	0.069
14d	Sham/CIBP	♀	<.001***	0.143
17d	Naive/Sham	♀	0.413	0.822
17d	Naive/CIBP	♀	<.001***	0.055
17d	Sham/CIBP	♀	<.001***	0.040
Pre	Naive/Sham	♂	0.631	0.998
Pre	Naive/CIBP	♂	0.981	0.997
Pre	Sham/CIBP	♂	0.857	0.980
3d	Naive/Sham	♂	0.905	0.982
3d	Naive/CIBP	♂	0.178	0.750
3d	Sham/CIBP	♂	0.180	0.807
6d	Naive/Sham	♂	0.223	0.761
6d	Naive/CIBP	♂	<.001***	0.192
6d	Sham/CIBP	♂	<.001***	0.213
8d	Naive/Sham	♂	0.426	0.766
8d	Naive/CIBP	♂	0.031*	0.630
8d	Sham/CIBP	♂	<.001***	0.191
14d	Naive/Sham	♂	0.619	0.917
14d	Naive/CIBP	♂	<.001***	0.181
14d	Sham/CIBP	♂	<.001***	0.237
17d	Naive/Sham	♂	0.277	0.792
17d	Naive/CIBP	♂	<.001***	0.109
17d	Sham/CIBP	♂	<.001***	0.123

Table S2. Ethogram of rat behavior in the homecage system.

The observation time includes the two first night hours (6 pm to 8 pm) before (baseline) and days 3, 8, 14, and 17 after walker 256 cell inoculation.

Category	Behavior	Description
Pain Behaviors	Ipsilaterlal Grooming	Grooming of ipsilateral hindleg
Grooming behavior	Self Grooming	Grooming, sniffing, licking body
Ambulatory Behaviors	Bipedal Stance	Bipedal stance, fully erect posture
	Jumping	Jump to the 1st floor
Resting Behaviors	Individual Resting	Rest without body contact with the cagemate
	Social Resting	Rest with body contact with the cagemate
	Total Resting	Total rest without consideration of the cagemate
Social Behavior	Playing	Soliciting, chasing, boxing, pinning
	Allo Grooming	Grooming, sniffing, licking body of cagemate
	Approximation	Approximation, Head length, purposeful

Table S3. Cohen's κ values of home cage observation analysis.

Behavior	Cohen's κ
Individual Resting	0.929
Social Resting	0.965
Bipedal Stance	0.943
Jump to the 1st floor	0.957
Food Ingestion	0.874
Self Grooming	0.967
Playing	0.784
Allo Grooming	0.898
Ipsilateral Grooming	0.841
Food Ingestion	0.874
Approximation	0.974

Table S4. Cluster analysis of PCA results (Figure S4) by multivariate analysis of variance (MANOVA); p-values: *<0.05, **<0.01, *<0.001.**

Time	Model	♀/♂	Sig	Wilks/Lambda
Pre	Naive-Naive/Sham-Sham	♀	0.975	0.850
Pre	Naive-Naive/CIBP-CIBP	♀	0.874	0.217
Pre	Naive-Naive/CIBP-ShamBY	♀	0.92	0.686
Pre	Naive-Naive/ShamBY-CIBP	♀	0.741	0.26
Pre	Sham-Sham/CIBP-CIBP	♀	0.078	0.362
Pre	Sham-Sham/ CIBP-ShamBY	♀	0.382	0.807
Pre	Sham-Sham/ ShamBY-CIBP	♀	0.053	0.521
Pre	CIBP-CIBP/ CIBP-ShamBY	♀	0.392	0.812
Pre	CIBP-CIBP/ ShamBY-CIBP	♀	0.406	0.818
Pre	CIBP-ShamBY/ ShamBY-CIBP	♀	0.86	0.941
3d	Naive-Naive/Sham-Sham	♀	0.398	0.868
3d	Naive-Naive/CIBP-CIBP	♀	0.222	0.793
3d	Naive-Naive/CIBP-ShamBY	♀	0.815	0.955
3d	Naive-Naive/ShamBY-CIBP	♀	0.691	0.921
3d	Sham-Sham/CIBP-CIBP	♀	0.102	0.704
3d	Sham-Sham/ CIBP-ShamBY	♀	0.508	0.86
3d	Sham-Sham/ ShamBY-CIBP	♀	0.299	0.765
3d	CIBP-CIBP/ CIBP-ShamBY	♀	0.491	0.854
3d	CIBP-CIBP/ ShamBY-CIBP	♀	0.405	0.818
3d	CIBP-ShamBY/ ShamBY-CIBP	♀	0.963	0.985
8d	Naive-Naive/Sham-Sham	♀	0.35	0.851
8d	Naive-Naive/CIBP-CIBP	♀	0.197	0.639
8d	Naive-Naive/CIBP-ShamBY	♀	0.536	0.719
8d	Naive-Naive/ShamBY-CIBP	♀	0.209	0.538
**8d**	**Sham-Sham/CIBP-CIBP**	**♀**	**0.002***	**0.379**
**8d**	**Sham-Sham/ CIBP-ShamBY**	**♀**	**0.043***	**0.497**
**8d**	**Sham-Sham/ ShamBY-CIBP**	**♀**	**0.003***	**0.28**
8d	CIBP-CIBP/ CIBP-ShamBY	♀	0.996	0.999
8d	CIBP-CIBP/ ShamBY-CIBP	♀	0.435	0.831
8d	CIBP-ShamBY/ ShamBY-CIBP	♀	0.46	0.733
17d	Naive-Naive/Sham-Sham	♀	0.554	0.913
17d	Naive-Naive/CIBP-CIBP	♀	0.532	0.908
17d	Naive-Naive/CIBP-ShamBY	♀	0.114	0.618
**17d**	**Naive-Naive/ShamBY-CIBP**	**♀**	**0.033***	**0.469**
17d	Sham-Sham/CIBP-CIBP	♀	0.234	0.804
**17d**	**Sham-Sham/ CIBP-ShamBY**	**♀**	**0.043***	**0.496**
**17d**	**Sham-Sham/ ShamBY-CIBP**	**♀**	**0.007***	**0.333**
17d	CIBP-CIBP/ CIBP-ShamBY	♀	0.329	0.781
17d	CIBP-CIBP/ ShamBY-CIBP	♀	0.266	0.745
17d	CIBP-ShamBY/ ShamBY-CIBP	♀	0.802	0.915
**Time**	**Model**	**♀/♂**	**Sig**	**Wilks/Lambda**
**Pre**	**Naive-Naive/Sham-Sham**	**♂**	**0.035***	**0.52**
**Pre**	**Naive-Naive/CIBP-CIBP**	**♂**	**0.002***	**0.382**
Pre	Naive-Naive/CIBP-ShamBY	♂	0.261	0.742
**Pre**	**Naive-Naive/ShamBY-CIBP**	**♂**	**0.011***	**0.37**
Pre	Sham-Sham/CIBP-CIBP	♂	0.980	0.997
Pre	Sham-Sham/ CIBP-ShamBY	♂	0.113	0.616
Pre	Sham-Sham/ ShamBY-CIBP	♂	0.517	0.864

**Pre**	**CIBP-CIBP/ CIBP-ShamBY**	♂	**0.012***	**0.376**
Pre	CIBP-CIBP/ ShamBY-CIBP	♂	0.391	0.812
Pre	CIBP-ShamBY/ ShamBY-CIBP	♂	0.08	0.364
**3d**	**Naive-Naive/Sham-Sham**	♂	**<.001*****	**0.34**
**3d**	**Naive-Naive/CIBP-CIBP**	♂	**<.001*****	**0.299**
3d	Naive-Naive/CIBP-ShamBY	♂	0.218	0.713
3d	Naive-Naive/ShamBY-CIBP	♂	0.096	0.685
3d	Sham-Sham/CIBP-CIBP	♂	0.126	0.726
**3d**	**Sham-Sham/ CIBP-ShamBY**	♂	**0.002****	**0.256**
**3d**	**Sham-Sham/ ShamBY-CIBP**	♂	**<.001*****	**0.2**
**3d**	**CIBP-CIBP/ CIBP-ShamBY**	♂	**<.001*****	**0.176**
**3d**	**CIBP-CIBP/ ShamBY-CIBP**	♂	**0.001*****	**0.222**
3d	CIBP-ShamBY/ ShamBY-CIBP	♂	0.432	0.399
8d	Naive-Naive/Sham-Sham	♂	0.735	0.954
**8d**	**Naive-Naive/CIBP-CIBP**	♂	**0.001*****	**0.365**
**8d**	**Naive-Naive/CIBP-ShamBY**	♂	**0.015***	**0.393**
**8d**	**Naive-Naive/ShamBY-CIBP**	♂	**<.001*****	**0.188**
**8d**	**Sham-Sham/CIBP-CIBP**	♂	**<.001*****	**0.34**
**8d**	**Sham-Sham/ CIBP-ShamBY**	♂	**0.011***	**0.368**
**8d**	**Sham-Sham/ ShamBY-CIBP**	♂	**<.001*****	**0.138**
**8d**	**CIBP-CIBP/ CIBP-ShamBY**	♂	**<.001*****	**0.19**
**8d**	**CIBP-CIBP/ ShamBY-CIBP**	♂	**<.001*****	**0.068**
8d	CIBP-ShamBY/ ShamBY-CIBP	♂	0.072	0.35
17d	Naive-Naive/Sham-Sham	♂	0.528	0.906
**17d**	**Naive-Naive/CIBP-CIBP**	♂	**<.001*****	**0.343**
**17d**	**Naive-Naive/CIBP-ShamBY**	♂	**0.005****	**0.308**
**17d**	**Naive-Naive/ShamBY-CIBP**	♂	**0.272**	**0.272**
**17d**	**Sham-Sham/CIBP-CIBP**	♂	**0.319**	**0.319**
**17d**	**Sham-Sham/ CIBP-ShamBY**	♂	**0.368**	**0.368**
**17d**	**Sham-Sham/ ShamBY-CIBP**	♂	**0.435**	**0.435**
**17d**	**CIBP-CIBP/ CIBP-ShamBY**	♂	**0.154**	**0.154**
**17d**	**CIBP-CIBP/ ShamBY-CIBP**	♂	**0.178**	**0.178**
17d	CIBP-ShamBY/ ShamBY-CIBP	♂	0.531	0.531

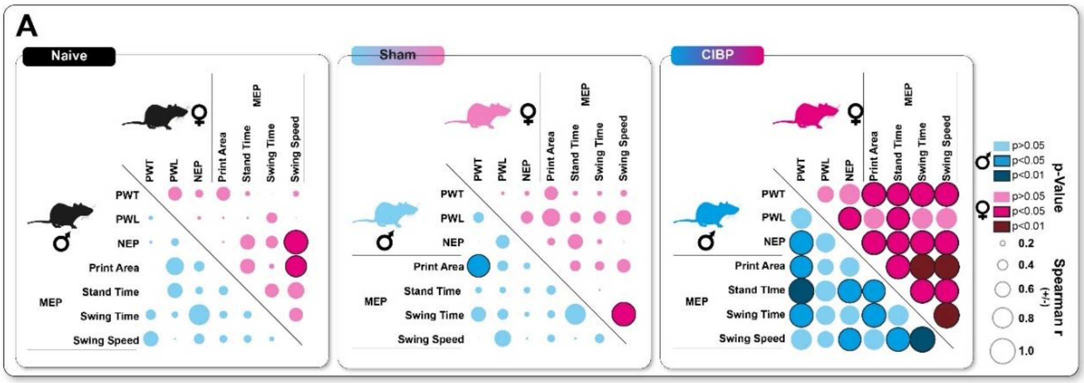

B

		PWT	PWL	NEP	Area	Stand	Swing Time	Swing Speed	BW
Naive	PWT		0.55	-0.32	-0.55	0.2	0.03	0.24	-0.26
	PWL	0.17		0.14	0.09	0.09	-0.43	-0.03	-0.66
	NEP	-0.12	-0.31		-0.09	0.6	0.49	**-0.94**	0.2
	Print Area	-0.03	0.71	0.42		-0.6	0.2	**-0.84**	-0.09
	Stand Time	0.03	0.6	-0.31	0.37		0.55	-0.66	0.03
	Swing Time	0.41	-0.26	0.83	0.31	0.31		-0.55	0.03
	Swing Speed	-0.61	-0.08	0.42	0.31	0.31	0.2		0.15
	Body Weight	-0.55	0.09	-0.71	0.49	0.49	**-0.88**	0.2	
Sham	PWT		-0.14	0.2	0.54	-0.2	0.2	-0.2 6	0.14
	PWL	0.43		-0.49	0.71	-0.43	0.49	-0.6	-0.43
	NEP	0.03	0.54		-0.31	0.6	0.2	0.08	-0.49
	Print Area	**0.89**	0.43	-0.26		-0.43	0.37	-0.54	-0.14
	Stand Time	-0.26	-0.26	-0.26	-0. 37		0.08	0.03	0.71
	Swing Time	-0.6	-0.43	0.2	-0.77	0.83		**-0.94**	-0.09
	Swing Speed	0.03	0.66	-0.09	0.26	-0.09	-0.37		-0.03
	Body Weight	-0.66	-0.77	-0.71	-0.49	0.43	0.6	-0.43	
CIBP	PWT		0.66	0.83	**0.94**	**0.89**	**-0.94**	**0.94**	**-0.94**
	PWL	0.83		**0.89**	0.83	**0.89**	-0.83	0.83	-0.83
	NEP	**0.94**	0.77		**0.94**	**0.89**	**-0.94**	**0.94**	**-0.94**
	Print Area	**0.89**	0.65	0.77		**0.94**	**1.00**	**-1.00**	**-1.00**
	Stand Time	**1.00**	0.83	**0.94**	**0.89**		**-0.94**	**0.94**	**-0.94**
	Swing Time	**0.94**	0.77	-0.77	**0.94**	-0.83		**-1.00**	**1.00**
	Swing Speed	-0.83	-0.66	**0.89**	-0.83	**0.94**	**1.00**		**-1.00**
	Body Weight	**-1.00**	-0.83	**-0.94**	**-0.89**	**-1.00**	**-0.94**	**0.93**	

♂ p>0.05 / p<0.05 / p<0.01 ♀ p>0.05 / p<0.05 / p<0.01

Figure S1. Correlation matrix between pain-related behavior features (A) The circle size represents the strength of the correlation; black cycle borders display significant correlation (Spearman r). (B) Representation of the raw Spearman correlations. Opaque colors represent significant correlations (<0.05).

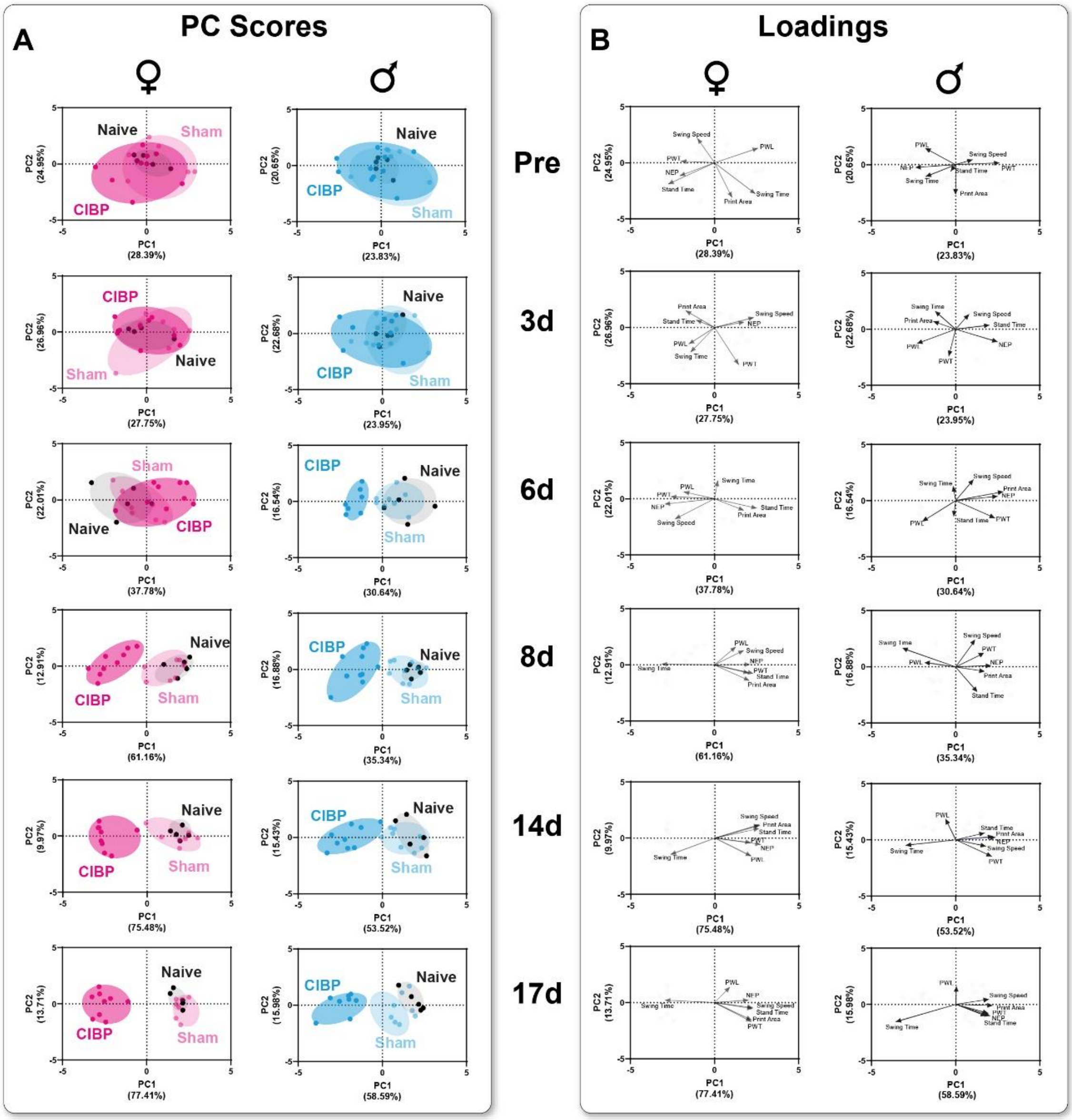

Figure S2. Bone cancer causes group segregation based on pain-related behavior trajectories in both sexes of rats. (A) Principal component analysis (PCA) of multi-feature behavioral data was applied to identify a CIBP-induced phenotype in a time-dependent manner. Significant group segregation was determined at 6d for CIBP males and 8d for CIBP females. (B) Representation of the representative loadings of the individual components within the PCA analysis. The PC components were selected to determine the eigenvalues. MANOVA was used for cluster analysis in PCA.

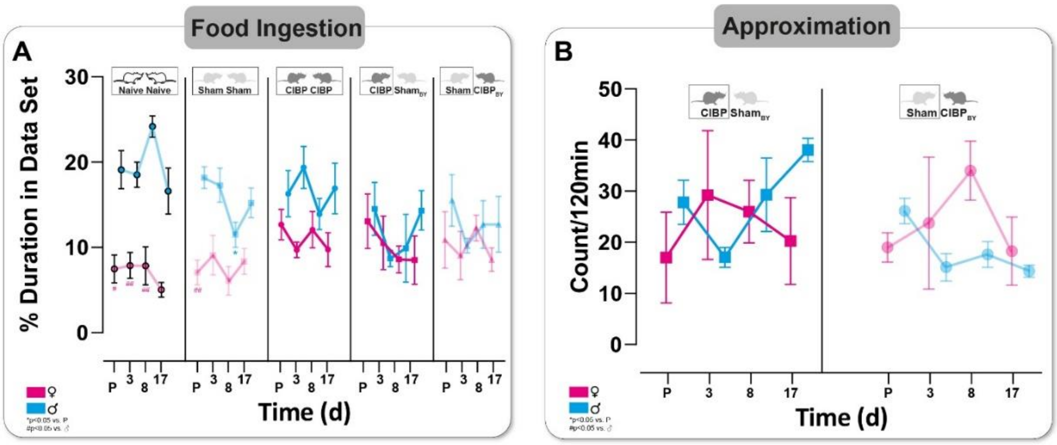

Figure S3. Bone cancer does not affect the rat's feed intake and approximation of both sexes. (A) Duration of feed intake in all four different housing conditions and (B) frequency of cage mate approximation in CIBP-ShamBY housing combination. Each housing combination was repeated four times with other animals (N=8, naive; N=8 sham; N=8 CIBP; N=4 CIBP-shamBY; N=4 shamBY-CIBP). Boxes represent the data shown. Results are expressed as mean± SEM. Two-way ANOVA (repeated measures based on GLM) followed by Dunnett's multiple comparison test * for comparison to sham; P- Values: * ≤ 0.05, ** ≤ 0.01, ***≤ 0.001. † for comparison to male; P-values: †≤ 0.05, ††≤ 0.01, †††≤ 0.001.

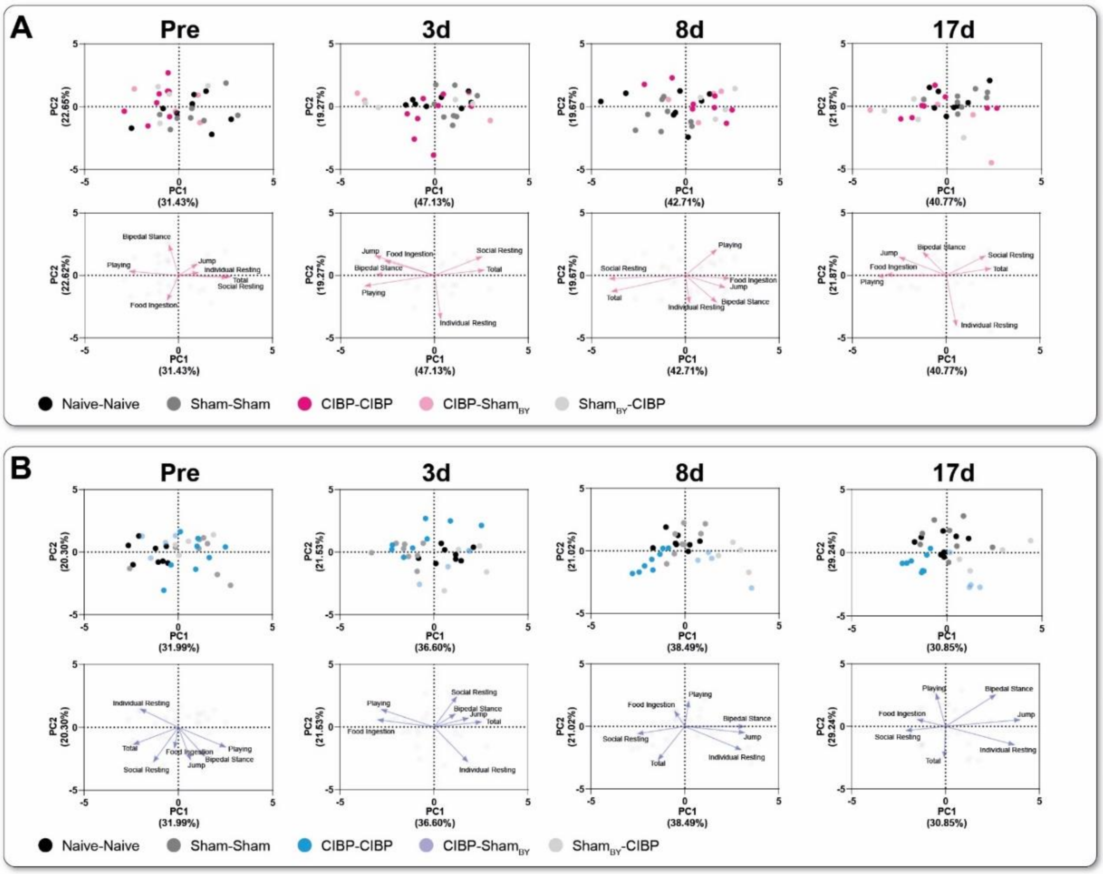

Figure S4. Bone cancer alters daily behavior in rats of both sexes. Principal component analysis (PCA) of multi-feature complex behavioral data was applied to identify a CIBP-induced phenotype in a time-dependent manner in (A) females and (B) males. The PC components were selected to determine the eigenvalues. MANOVA was used for cluster analysis in PCA (see supplementary Table 4).

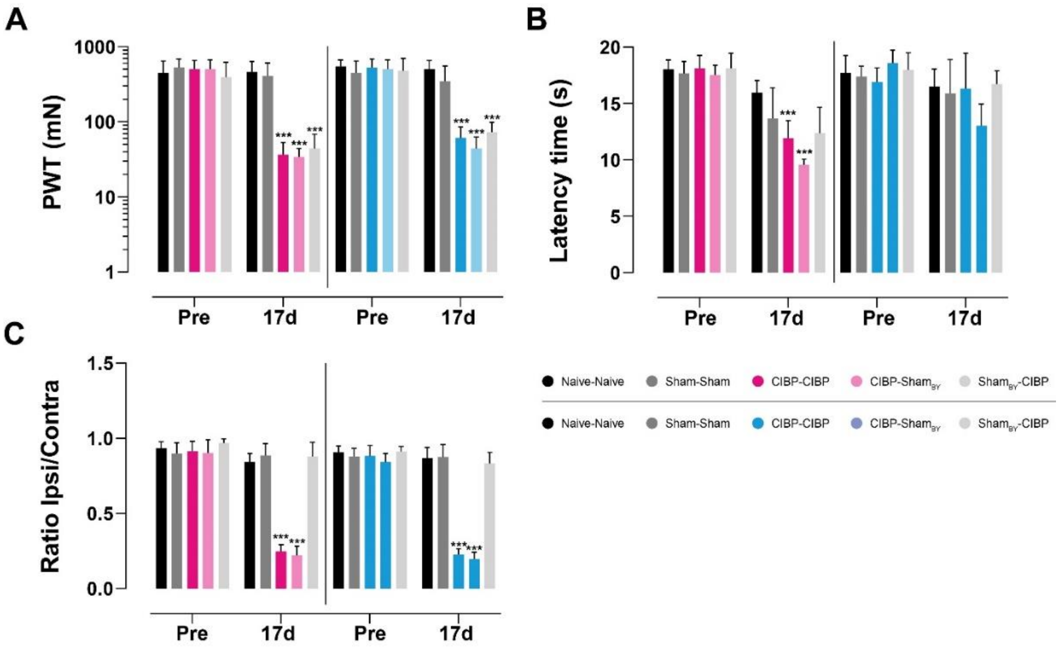

Figure S5. Social transfer of mechanical evoked pain-related behavior to sham bystander (BY) rats caused by cancer-induced bone pain in both sexes (A) Mechanical (PWT), heat (PWL) (B) thresholds, and (C) non-evoked pain-related behavior of the ipsilateral hind paws in rats of both sexes in four different housing conditions pre and 17d after cell inoculation. Four different housing combinations are shown here. Each housing combination was repeated four times with other animals (N=8, Naïve; N=8 sham; N=8 CIBP; N=4 CIBP-shamBY; N=4 shamBY-CIBP). Boxes represent the data shown. Results are expressed as mean± SEM. Two-way ANOVA (repeated measures based on GLM) followed by Dunnett's multiple comparison test * for comparison to sham; P- Values: * ≤ 0.05, ** ≤ 0.01, ***≤ 0.001.
